# PGAM5 expression and macrophage signatures in non-small cell lung cancer associated with chronic obstructive pulmonary disease (COPD)

**DOI:** 10.1186/s12885-018-5140-9

**Published:** 2018-12-10

**Authors:** F. Ng Kee Kwong, A. G. Nicholson, S. Pavlidis, I. M. Adcock, K. F. Chung

**Affiliations:** 1 Airways Disease, National Heart and Lung Institute, Imperial College London, London, UK; 2 Department of Histopathology, Royal Brompton & Harefield NHS Foundation Trust, London, UK; 3 Department of Histopathology, The Cotman Centre, Norfolk and Norwich University Hospital NHS Foundation Trust, Colney Lane, Norwich, NR4 7UB UK

**Keywords:** Mitochondrion, Lung cancer, PGAM5 macrophage, Patient survival

## Abstract

**Background:**

COPD patients are at increased risk of developing non-small cell lung carcinoma that has a worse prognosis. Oxidative stress contributes to carcinogenesis and is increased in COPD patients due to mitochondrial dysfunction. We determined whether mitochondrial dysfunction is a contributing factor to the reduced survival of COPD patients with non-small cell lung carcinoma (NSCLC).

**Methods:**

Using a transcriptomic database and outcome data of 3553 NSCLC samples, we selected mitochondrial-related genes whose levels in the tumour correlated with patient mortality. We further selected those genes showing a ≥ 2 fold expression in cancer compared to normal tissue. Cell-type specific expression of these proteins in lung tissue from NSCLC patients who were non-smokers or smokers with or without COPD (healthy smokers) was determined by immunohistochemistry. Gene set variation analysis was used in additional NSCLC datasets to determine the relative expression of specific macrophage transcriptomic signatures within lung cancer tissue.

**Results:**

The expression of 14 mitochondrial-related genes was correlated with patient mortality and these were differentially expressed between cancer and normal lung tissue. We studied further the expression of one of these genes, PGAM5 which is a regulator of mitochondrial degradation by mitophagy. In background lung tissue, PGAM5 was only expressed in alveolar macrophages, with the highest expression in smokers with COPD compared to healthy smokers and non-smokers. In cancerous tissue, only the malignant epithelial cells and associated macrophages at the periphery of the cancer expressed PGAM5. Pre-neoplastic epithelium also showed the expression of PGAM5. There was no difference in expression in cancer tissue between COPD, healthy smoker and non-smoker groups. Macrophages at the edge of the cancer from COPD patients showed a trend towards higher expression of PGAM5 compared to those from the other groups. There was a significant correlation between PGAM5 expression in cancer tissue and the level of expression of 9 out of 49 previously-defined macrophage transcriptomic signatures with a particular one associated with patient mortality (*p* < 0.05).

**Conclusion:**

PGAM5 is expressed in pre-neoplastic tissue and NSCLC, but not in normal epithelium. The association between PGAM5 expression and patient mortality may be mediated through the induction of specific macrophage phenotypes.

**Electronic supplementary material:**

The online version of this article (10.1186/s12885-018-5140-9) contains supplementary material, which is available to authorized users.

## Background

NSCLC presents with locally advanced or metastatic disease in 80% of cases [1, 2], which accounts for its dismal prognosis [3]. Although smoking is by far the most common risk factor for NSCLC, other risk factors include pulmonary fibrosis, tuberculosis, radiation therapy and COPD [4]. Up to 70% of lung cancer smokers have pre-existing COPD prior to cancer diagnosis [5, 6]. COPD patients are at an increased risk of developing lung cancer, irrespective of their smoking history [7–9]. Smokers with airflow obstruction have a five-fold increased risk of lung cancer compared to those with normal lung function [5]. As well as airflow obstruction, emphysema diagnosed on computed tomography (CT) is also another independent risk factor for lung cancer [10, 11]. The presence of COPD and/or emphysema are also predictors of reduced survival of NSCLC patients or increased risk of recurrence following surgery [12–15].

Oxidative stress is a well-recognised driver of carcinogenesis and is present in smokers and COPD patients, with the greatest degree of oxidative stress present in the airways of patients with COPD [16]. Reactive oxygen species produced in mitochondria from oxidative phosphorylation represent the main cellular source of oxidative stress, and increased production of mitochondrial ROS seen in airway epithelial cells of patients with COPD is associated with mitochondrial dysfunction [16]. Under increased oxidative stress, metabolically-active cells may undergo an increase in mitochondrial biogenesis and also in the degradation of damaged mitochondria within autolysosomes through the process of mitophagy. An inability to increase biogenesis and/ or induce mitophagy may lead to the accumulation of dysfunctional mitochondria and subsequently, increased oxidative stress [17].

The prognosis of lung cancer is also dependent on the immune inflammatory response within the tumour stroma [18]. One of the key components of the stroma is the tumour-associated macrophage which has traditionally been divided into the pro-inflammatory M1 phenotype and the anti-inflammatory M2 phenotype. The presence of M1 macrophages within the tumour islets of NSCLC may confer a survival advantage while the presence of M2 macrophages is a poor prognostic factor [19]. The effector functions of macrophages in the M1 and M2 states are dependent on changes in mitochondrial metabolism [20].

We therefore reasoned that mitochondrial dysfunction could be a driving mechanism underlying the increased propensity of COPD patients to develop cancer and the poor prognosis of NSCLC patients with COPD. In the current study, we demonstrated enhanced expression of the mitophagy-inducing protein PGAM5 in malignant epithelial cells, as well as in the alveolar macrophages from normal lung and adjacent to cancer and that their expression was related to the survival of non-small cell lung cancer patients. We also showed that the expression of PGAM5 was related to specific macrophage phenotypes. Our data suggests that the expression of PGAM5 in lung cancer is associated with specific macrophage phenotypes and patient survival.

## Methods

### Screening for mitochondrial-related candidate genes in lung cancer

The steps involved in this study are outlined in Table 1. More than 250 mitochondrial-related genes were selected by literature mining and from the Molecular Signatures Database (M8479) [21]. We determined whether a change in the expression of each tumour-expressed mitochondrial-related gene was associated with patient survival. To this end, we accessed the cancer gene expression and outcome data from the public domain, which have been assembled in the Precog database [https://precog.stanford.edu/] [22]. The latter contains transcriptomic and survival data of multiple cancer types, and specifically 3553 NSCLC (adenocarcinoma and squamous cell carcinoma) cases. In this database, the statistical associations between gene expression and clinical outcomes were assessed by z-scores, which are directly related to *p* values. Z-scores represent the number of standard deviations from the mean of a normal distribution. |z| > 1.96 is equivalent to a two-sided *p* < 0.05. After selecting for those genes with |z| > 1.96, we then examined the differential expression of genes between cancer and the normal surrounding tissue, using the publicly available RNA Seq Nexus database [23] [http://syslab4.nchu.edu.tw/] that includes transcriptomic data from 151 Stage 1 squamous cell carcinomas, 140 Stage 1 adenocarcinomas and 51 normal lung tissues. Further selection was made for genes showing a ≥ 2 fold expression in cancer compared to normal tissue.

**Table 1:** Stepwise approach to screen and assess in vivo functions of relevant mitochondrial-related genes in lung cancer

1. Mitochondrial genes	Selection of mitochondrial related genes using literature mining and molecular signatures database
2. Cancer outcome	Selection for genes showing correlation with the survival of cancer patients using a publicly available database
3. Differential gene expression in cancer	Selection for genes showing differential gene expression between cancer and normal lung tissue using a publicly available database
4. Cell specific protein expression	Demonstrate differential protein expression between cancer and normal tissue in vivo Show cell type specific expression of mitochondrial-related proteins by immunohistochemistry
5. Cellular phenotype	Correlate cell type specific expression with phenotype and cancer outcome using publicly available databases.

### Lung tissue cohort characteristics

Formalin-fixed paraffin-embedded tissue, surplus to diagnostic purposes, was obtained from patients undergoing lung cancer resection. As well as cancer tissue, there was background lung tissue, from the same lobe as the cancer, which pathologists routinely sample to detect any co-existing histological abnormalities. Patients’ medical records, lung function tests and imaging reports were reviewed. Informed consent was obtained from the donor prior to surgery for use of surgically-excised tissues for research purposes. This study was approved by the Health Research Authority, South Central – Hampshire B Research Ethics Committee (REC Reference: 15/SC/0569). The lung cancer patients had not previously undergone radiotherapy or chemotherapy. The staging of lung cancer was based on the 7th Lung cancer TNM classification and staging system. The patients were divided into non-smoking and smoking groups. The smokers were further subdivided according to their lung function tests, with the healthy smokers group defined by an FEV1/ FVC ratio greater than 70%. The COPD group was comprised of smokers with an FEV1/ FVC ratio less than 70%. In addition, the histological diagnosis of non-cancerous lung tissue from all patients were reviewed to determine the presence and severity of emphysema.

### Immunohistochemistry

Immunohistochemistry for PGAM5 was performed on cancer and background (‘normal’) tissue from lung cancer resections from non-smokers, healthy smokers and emphysema patients. Heat-mediated antigen retrieval was performed at pH 8.5. The PGAM5 (Abcam) antibody was applied at a 1:300 dilution. To detect the primary antibody, the Optiview DAB detection kit (Ventana Medical Systems) was used and included the secondary antibody. As negative controls, cancer and background lung tissue from the same patients were used, but the primary antibody was not added and was replaced by buffer instead.

Immunostaining was assessed using a semi-quantitative scoring system (H score) [24]. Briefly, 100 cells of interest (tumour or macrophages) were counted, and an H-score was generated by adding the percentage of strongly stained nuclei multiplied by 3, the percentage of moderately stained nuclei multiplied by 2, and the percentage of weakly stained nuclei multiplied by 1, giving a possible score of range of 0–300 [25].

### Gene set variation analysis

Gene set variation analysis (GSVA) was used to compare the expression of these macrophage signatures across the cancer datasets GSE31210 and GSE72194. GSVA allows for the calculation of sample-wise enrichment scores (ES) [26, 27]. We compiled 49 gene sets each related to a specific macrophage activation status obtained from Xue et al [28] and the ES was calculated for each gene set for each subject. Dataset GSE 31210 comprised of transcriptomic and outcome data for 226 primary Stage 1 and 2 lung adenocarcinomas [29]. Dataset GSE72194 [30] combined 5 previous datasets with the clinical and transcriptomic data of 338 adenocarcinomas and 294 squamous cell carcinomas.

### Statistical analysis

The immunohistochemical score (H score) was analysed using a Kruskal-Wallis test, with one-way ANOVA tests across the three groups of subjects and Dunn’s multiple comparison tests between the groups. ANOVA was used to analyse the ES differences among group means and the Student’s t-test was applied to compare the ES differences between the 2 means. *p* < 0.05 was considered statistically significant.

## Results

### Screening for mitochondrial-related genes in the pathogenesis of NSCLC

Fourteen mitochondrial-related genes were differentially expressed between cancer and normal tissue, as well as being associated with a change in survival of the patients with the magnitude of the Precog z score exceeding 1.96 (Table 2). The mitochondrial-related genes involved were related to the oxidative phosphorylation complex, mitophagy, glycolysis, necroptosis and anti-oxidants. With regards to mitophagy, its dysregulation in the pathogenesis of other cancers has previously been demonstrated [31, 32] and the Pink1-parkin mitophagy pathway is known to be altered in COPD [33, 34]. However, little is known of the role of PGAM5 in carcinogenesis and, to our knowledge, its role in NSCLC has not been previously studied. We therefore focused on PGAM5.

**Table 2:** Differentially-expressed mitochondrial-related genes between normal and cancer tissue and association with patient survival

	Squamous cell carcinoma	Fold change	Hazard Ratio	Adenocarcinoma	Fold change	Hazard ratio
Oxidative phosphorylation complex	Ndufs1	5	1.5	Ndufs1	4	1.6
	Ndufv1	2.9	1.1	Ndufa9	2.4	1.8
	Bcs1L	7	1.2	ATP5G1	7.5	3.2
	ATP5i	4.8	1.4	ATP5J2	3.7	2.3
Mitophagy	PGAM 5	2.5	3.1			
Glycolysis				ENO 1	2	2.7
				ENO2	3.5	1.9
				Aldo A	2.6	7.3
Necroptosis	FADD	2.2	2.3			
Anti-oxidants				GPX 2	74	1.2
Autophagy				LRPPRC	3	1.7

The differential expression of genes between cancer and the normal surrounding tissue was obtained from the publicly-available RNA Seq Nexus database [17] [http://syslab4.nchu.edu.tw/]. The genes with ≥2 fold change of expression in cancer versus normal tissue expression were selected, (all at *p* < 0.05). The effect of gene expression on patient outcome was also assessed using the Precog database. The genes shown are associated with increased mortality of NSCLC patients, with the hazard ratio shown in Precog

### Patient characteristics

The demographic characteristics of the patients are shown in Table 3. As expected, the FEV1/ FVC ratio, % predicted FEV1 and % predicted FEV1/ FVC were lower in the COPD group, compared to the other 2 groups (non-smokers and healthy smokers). There was no difference in the smoking index between the 2 groups of smokers. There was a predominance of adenocarcinomas in the non-smoking population, unlike in the other 2 groups (*p* < 0.05). However, there was no difference in the proportion of Stage 1, Stage 2 and Stage 3 cancer across the 3 groups of patients.

**Table 3:** Characteristics of patients from whom cancer tissue was obtained

	Non-smoker	Healthy Smoker	COPD	*p* value
n	7	11	11	NS
Age (years)	73.9 ± 10.5	65.4 ± 2.4	69.6 ± 2.1	NS
Male (% group)	40	46	62	NS
Smoking index (pack-year)	N/A	51 ± 17.4	66 ± 21	NS
FEV1	1.9 ± 0.4	2.3 ± 0.2	2.0 ± 0.2	NS
% predicted FEV1	89.2 ± 3.3	87.8 ± 3.6	77.9 ± 4.7	< 0.05
FVC	2.3 ± 0.7	3.0 ± 0.2	3.5 ± 0.4	< 0.05
% predicted FVC	103.8 ± 5.8	95.9 ± 5.9	103.5 ± 2.6	NS
FEV1/ FVC (%)	76.3 ± 9.8	76.1 ± 1.3	58.3 ± 2.9	< 0.05
Emphysema present / Emphysema status known	0/7	5/10	8/10	< 0.05
Cancer Histology	6 ADC, 1 SCC	5 ADC, 6 SCC	2 ADC, 8 SCC, 1 NSCLC	< 0.05
Cancer Stage	3 Stage1 4 Stage2	8 Stage1 3 Stage 2	8 Stage 1 2 Stage 2 1 Stage 3	NS

Kruskal-Wallis test was used, with one-way ANOVA tests across the three groups of subjects and Dunn’s multiple comparison tests between the groups. NS = not significant (*p* > 0.05)

*ADC* adenocarcinoma, *SCC* squamous cell carcinoma, *NSCLC* non small cell carcinoma not otherwise specified. Emphysema status was determined by histology of non-cancerous lung from the same lobe as the cancer

We assessed emphysema from the histology of the non-cancerous lung tissue from the same lung lobe as the cancer in 27 patients (out of total of 29 patients). All 7 non-smokers did not have emphysema. There were 11 COPD patients, of which the emphysema status could not be determined in one patient owing to the required histology not being available. Of the remaining 10 COPD patients, 8 had emphysema and 2 did not have emphysema. Of the 11 healthy smokers, the emphysema status could not be determined in one patient for the same reason as above. Five healthy smokers were found to have emphysema with an equal number in this group with no emphysema.

### Immunohistochemistry

**Fig. 1:**
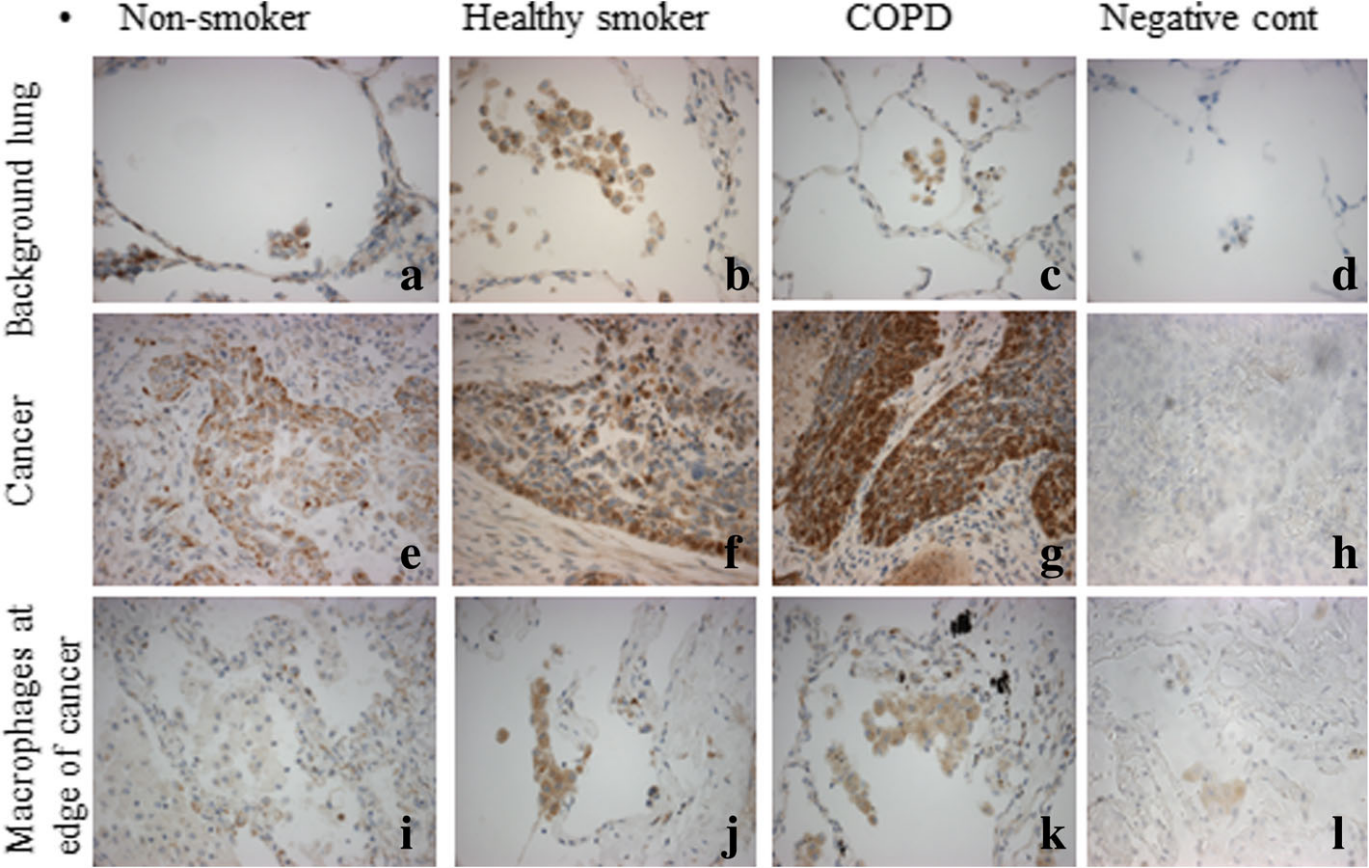
Immunohistochemistry for PGAM 5 expression in background lung (**a**-**d**), squamous cell carcinoma (**e**-**h**) and alveolar macrophages at the edge of cancer (**i**-**l**). Magnification ×40. The first row shows background lung tissue with alveolar walls and macrophages in the alveolar space. PGAM 5 is expressed by alveolar macrophages (only cell type showing brown staining in this row), with higher expression in healthy smokers and COPD groups, compared to non-smokers. The second row shows cancerous tissue with epithelial cells and stromal cells. PGAM 5 is expressed by the malignant epithelial cells (showing brown staining), but not stroma, in all three groups of patients. There was no difference in PGAM5 expression by the epithelial cells across the 3 groups of patients. The third row shows alveolar spaces at the edge of cancerous tissue. PGAM5 is expressed in the alveolar macrophages (only cell type showing brown staining in this row)

**Fig. 2:**
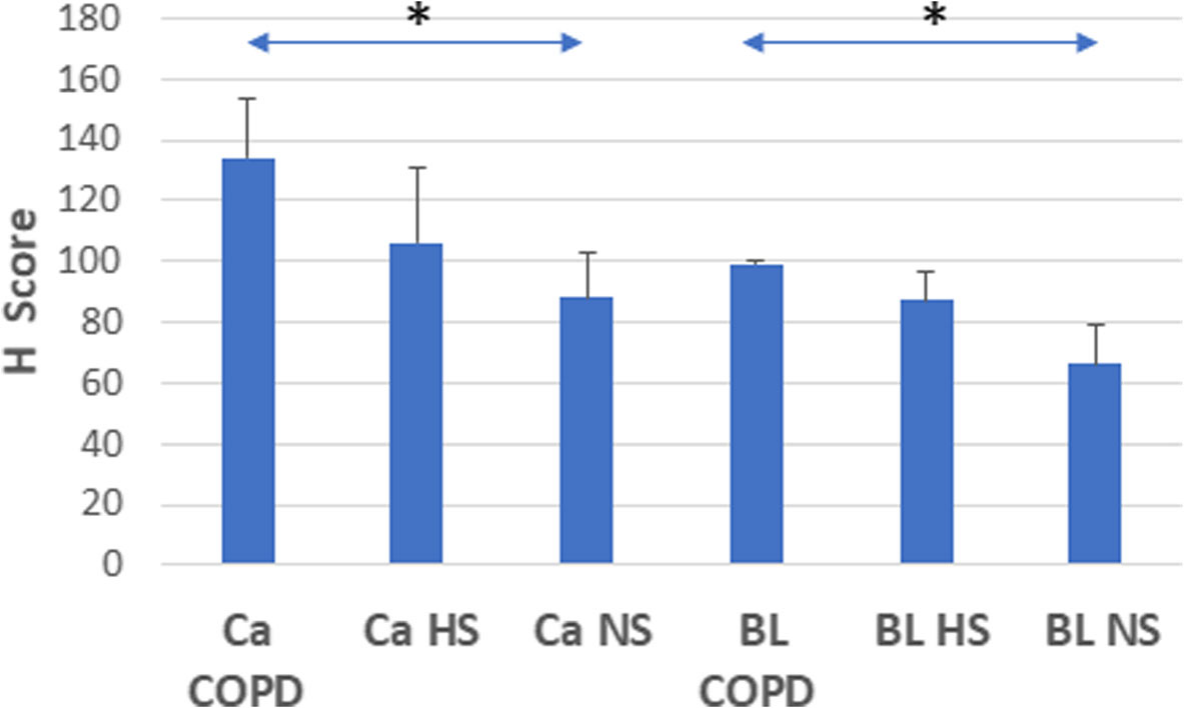
PGAM5 expression in alveolar macrophages at the edge of cancer (Ca) and in background lung tissue (BL). Ca COPD: Lung cancer tissue from patients with COPD; Ca HS: Lung cancer tissue from healthy smokers; Ca NS: Lung cancer tissue from non-smokers. BL COD: Background lung tissue from patients with COPD; BL HS: Background lung tissue from healthy smokers and BL NS: Background lung tissue from non-smokers. The expression of PGAM5 in alveolar macrophages was highest in the COPD group, compared to healthy smokers and non-smokers. However, there was no detectable difference in expression between the healthy smokers and COPD patients. *, *p* < 0.05

Immunohistochemistry was performed on background lung and cancerous tissue from 3 groups of NSCLC patients: non-smokers; healthy smokers and smokers with COPD. In background lung tissue, PGAM5 was only expressed in alveolar macrophages (Fig. 1). Although the number of alveolar macrophages was increased in the healthy smokers (4-fold increase) and COPD patients (5-fold increase) compared to the non-smoker group, there was a quantitative increase in PGAM5 expression per macrophage (Fig. 2). There was no expression in alveolar or bronchial epithelial cells in any of the subject groups. The expression of PGAM5 in alveolar macrophages was highest in the COPD group, compared to healthy smokers and non-smokers (Figs. 1, 2). PGAM5 expression in the alveolar macrophages was also higher in background lung from the 13 smokers with emphysema (H score, 95 ± 3.7, mean and SEM) compared to all patients without emphysema (smokers and non-smokers; 65 ± 13, *p* < 0.05).

**Fig. 3:**
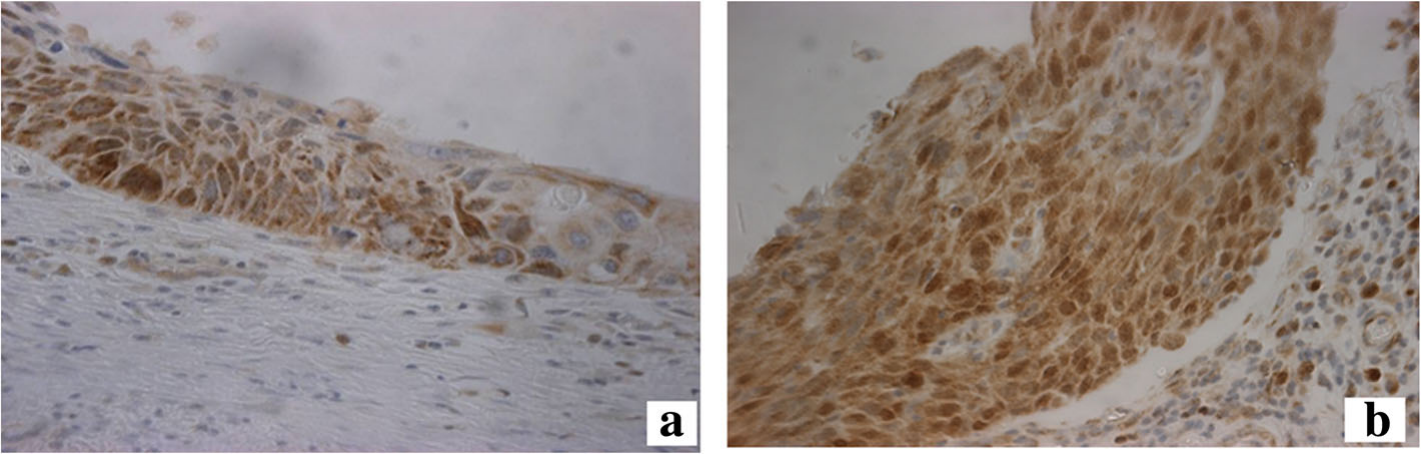
PGAM5 expression in pre-neoplastic epithelium (*n* = 6). Squamous dysplasia (**a**) and full thickness dysplasia, carcinoma in situ (**b**). There is expression of PGAM5 (indicated by brown staining in cells) in the epithelium of squamous dysplasia and carcinoma in situ

In cancerous tissue, only the malignant epithelial cells and alveolar macrophages at the periphery of the cancer expressed PGAM5 (Fig. 1). PGAM5 was expressed by the malignant epithelial cells of all tumours (*n* = 29), whether they were adenocarcinomas or squamous cell carcinoma and in pre-neoplastic epithelium (squamous dysplasia and carcinoma in situ) (Fig. 3). However, there was no expression within atypical adenomatous hyperplasia, a precursor of adenocarcinoma. There was no difference in PGAM5 expression by the epithelial cells across the 3 groups of patients, even if the comparison was made for either adenocarcinoma or squamous cell carcinoma cases separately across the three groups of patients. In addition, examining PGAM5 expression in tumour cells in all the smokers (whether healthy smokers or COPD) according to the degree of emphysema severity, we found no difference in expression between those without emphysema (H score, 168 ± 51; *n* = 7) and those with mild emphysema (H score, 151 ± 18; *n* = 6). Smokers with moderate emphysema showed a lower PGAM 5 expression (H score, 62.5 ± 18; *n* = 4) compared to smokers without emphysema or with mild emphysema (*p* < 0.05).

### Macrophage phenotype in NSCLC

**Fig. 4:**
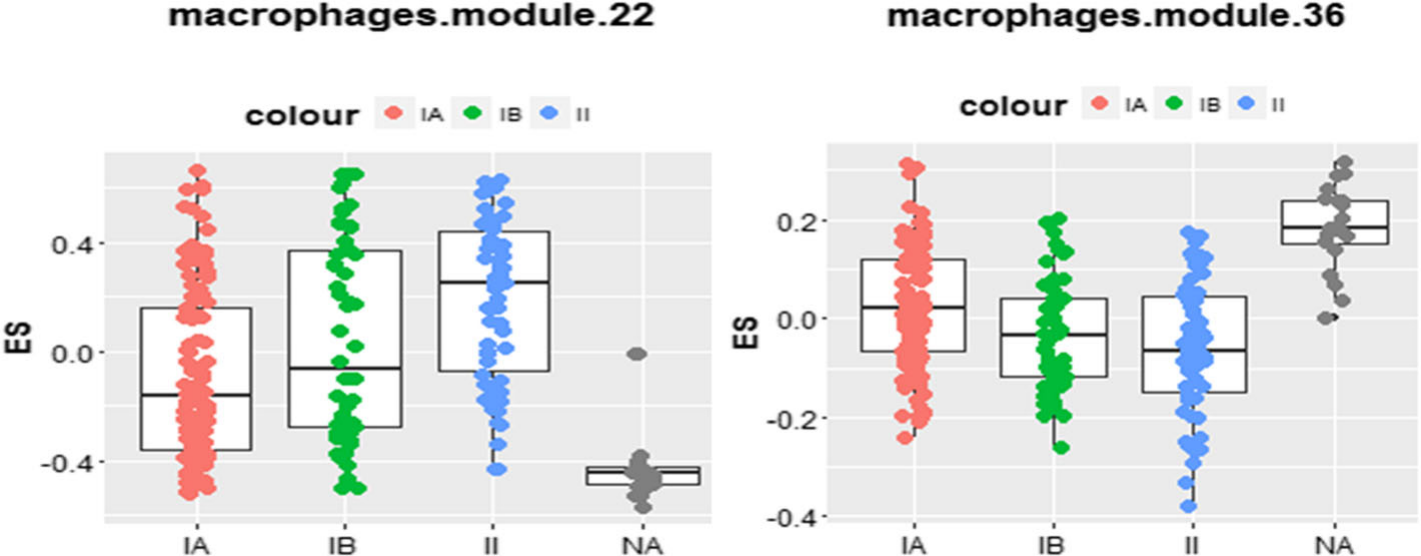
Expression of macrophage signatures in NSCLC, according to stage of cancer. GSVA was used to compare the expression of macrophage signatures across the cancer datasets GSE31210 and GSE72194. GSVA calculates sample-wise enrichment scores (ES). We compiled 49 gene sets each related to a specific macrophage activation status obtained from Xue et al. [13] and the ES was calculated for each gene set for each subject. NA, normal tissue. Two macrophage signatures (modules 22 and 36 which are similar to signatures of monocytes activated by oleic acid and tumour necrosis factor + Pam3CSK4 + prostaglandin E2 respectively) showed differential expression between normal lung tissue and Stages 1 and 2 of lung cancer (*p* < 0.05)

We first determined whether there was a correlation between the different macrophage signatures and clinical outcome in lung cancer. The dataset GSE 31210 comprised transcriptomic and clinic-pathological data of 226 primary Stage 1 and 2 lung adenocarcinomas and background lung tissue [29]. Using GSVA, the enrichment scores of two macrophage signatures [modules 22 and 36 which are similar to signatures of monocytes activated by oleic acid and tumour necrosis factor + Pam3CSK4 + prostaglandin E2, respectively] were correlated with the overall survival of patients (*p* < 0.05), as well as with differential expression between normal lung tissue and Stages 1 and 2 of lung cancer (*p* < 0.05) (Fig. 4). The genes comprising the relevant macrophage signatures are included in the Additional file 1: Table S1, which has been adapted from data from Xue et al [28].

Next, we studied whether PGAM5 expression was correlated with the macrophage phenotype present in tumour tissue in NSCLC dataset GSE72194 [30]. There was a positive correlation of PGAM5 expression with one macrophage signature (‘module 49’) (*r* = 0.3; *p* < 0.05) [Additional file 1: Table S1]. There was a negative correlation with 8 macrophage signatures: ‘modules 7, 9, 10, 11, 28, 36, 39 and 42’ [similar to signatures of monocytes activated by palmitic acid or IFN γ; IFN γ + Tumour Necrosis Factor; IL4 × 2; oleic acid; tumour necrosis factor + Pam3CSK4 + prostaglandin E2 × 2; prostaglandin E2 respectively], including ‘module 36’ (*r* = − 0.44 to − 0.24) (*p* < 0.05) but not ‘module 22’ [Additional file 1: Table S1]. Therefore, one macrophage signature (‘module 36’), which is correlated with PGAM5 expression, is also associated with the outcome of the cancer patients.

## Discussion

COPD patients are at increased risk of NSCLC while patients with established NSCLC have a worse outcome if they have concomitant COPD [5–9, 12–15]. Oxidative stress is an initiator of carcinogenesis [35] and the prognosis of NSCLC is partly dependent on the metabolic state of its tumour-associated macrophages [36]. Both the increased oxidative stress in COPD and the metabolic state of macrophages are dependent on the state of the mitochondrion [16, 20]. We therefore reasoned that there would be an altered expression of selective mitochondrial-related genes within tumour tissue and that this would be correlated with a change in patient survival. Fourteen mitochondrial-related genes showed a ≥ 2 fold difference in expression between NSCLC and normal lung tissue and their expression was correlated with a reduction in patient survival. Although dysfunction in mitophagy has previously been demonstrated in COPD and NSCLC, there have been no published studies of the mitophagy-related protein, PGAM5, in the pathogenesis of COPD and NSCLC.

PGAM5 functions in multiple cell death pathways [37] and regulates mitophagic protection against cell necroptosis [38]. PGAM5 catalyses the dephosphorylation of FUNDC1 which enhances its interaction with microtubule-associated protein 1A/1B-light chain 3, leading to mitophagy [39]. PGAM5 also promotes inflammasome activation in macrophages [40]. We report for the first time the expression of PGAM5 in lung tissue from COPD and NSCLC patients. There was 2.5 fold increase in PGAM5 mRNA expression in cancer tissue compared to background/ normal lung tissue. Using immunohistochemistry, the tumour cells but not the normal tissue expressed PGAM5. This confirmed that both mRNA and protein expression of PGAM5 are higher in cancer tissue than in normal tissue, although it is difficult to quantify the degree of increase in protein expression***.*** PGAM5 was consistently expressed in the malignant epithelial cells of lung cancer patients, with no difference in the level of expression across the 3 groups of patients, even if adenocarcinoma or squamous cell carcinoma cases were considered separately across the groups. The level of expression of PGAM5 in lung cancer itself cannot therefore explain why patients with COPD and NSCLC have the worse outcome. However, PGAM5 expression in carcinomas from smokers with moderate emphysema showed lower expression compared to those from smokers with mild or no emphysema. Although the significance of this finding may be limited by the small number of patients in the moderate emphysema group, this may reflect reduced mitophagy in this group of patients, compared to those with mild or no emphysema. This also points to the need of subclassifying patients according to emphysema severity in future studies. PGAM5 is also expressed in alveolar macrophages at the edge of the cancer.

Benign bronchial and alveolar epithelial cells do not express PGAM5 while pre-neoplastic epithelium such as squamous dysplasia and carcinoma in situ express PGAM5. The association of PGAM5 expression with worse prognosis in squamous cell carcinoma and its expression in the sequential transformation of squamous dysplasia into malignant squamous cells suggests a pathological role for PGAM5 in lung squamous cell carcinoma. The mechanisms by which PGAM5 may contribute to the malignant transformation of the epithelium is uncertain. It is possible that this may be related to the action of PGAM5 on cell death pathways, leading to uncontrolled cellular proliferation secondary to its overexpression.

PGAM5 expression was only detected within alveolar macrophages in non-cancerous lung tissue. The level of expression in alveolar macrophages in non-cancerous tissue was higher in smokers than non-smokers, with a trend towards highest expression in COPD or emphysematous patients. The phenotype of alveolar macrophages is altered in COPD [41] and the increased oxidative stress, resulting from excess free radicals in cigarette smoke, leads to mitochondrial damage and increased turnover, including mitophagy. This may be reflected by the increased expression of PGAM5 in alveolar macrophages. Increased mitochondrial turnover may also lead to a change in the metabolic phenotype of the alveolar macrophages which is known to be related to their immunomodulatory phenotypes [20]. PGAM5 in the alveolar macrophages may also lead to the activation of the inflammasome, which is important in triggering the inflammatory response in COPD [40].

In GSE 72194, PGAM5 expression within tumours was correlated with 9 out of 49 macrophage signatures (‘modules 7, 9, 10, 11, 28, 36, 39, 42, 49’) obtained from a study of macrophage molecular phenotypes by Xue et al. [28]. In GSE 31210, PGAM5 expression was negatively correlated with the macrophage ‘module 36’ (similar to signature of monocytes activated by tumour necrosis factor + Pam3CSK4 + prostaglandin E2) expression in lung cancer, which itself is inversely associated with mortality. This may partly explain our original observation that PGAM5 expression is associated with a worse outcome in NSCLC. We speculate that products from macrophages stimulated by PGAM5 may affect epithelial cell function and may account, at least in part, for the increased risk of lung cancer in patients with COPD. Further work is needed to elucidate the relevant mechanisms.

The findings of this study are limited by the modest number of cases we have analysed with immunohistochemistry, findings that would need to be confirmed in a larger study. Another limitation relates to the potential differences in tumour stroma content between the different tumour samples within the datasets used. To quantify RNA expression within the tumour cells and not stroma, laser capture microdissection of tumour cells would have to be performed. Although such data may be available from individual studies with small number of samples, large datasets mostly include data from studies in which laser capture tissue dissection was not carried out. However, our data on PGAM5 expression has been validated by immunohistochemistry with the demonstration of PGAM5 specifically in tumour cells and not in stromal cells. Variations of RNA expression due to intratumoural heterogeneity have been reduced by the relatively large number of tumour samples [151 squamous cell carcinomas and 140 adenocarcinomas] examined at an early stage of cancer (Stage 1).

Also, in our study, it was not possible to determine if there were macrophage signatures specific to COPD-associated cancer in comparison to non-COPD associated cancer. We propose that the tumour-associated macrophages in COPD-associated lung cancer are different from those in non-COPD associated cancer. This would account for the worse prognosis of the former, as we have shown that specific macrophage phenotypes are associated with prognosis.

## Conclusion

In summary, the expression of specific mitochondrial-related proteins and macrophage signatures are associated with the outcome of NSCLC. The expression of PGAM5 in lung cancer was correlated with specific macrophage phenotypes, one of which was associated with lung cancer mortality. PGAM5 is expressed in pre-malignant and malignant epithelial cells, but not in benign epithelium and may therefore play a role in the malignant transformation of airway epithelial cells.

## Additional file

Additional file 1: **Table S1.** Gene signatures of relevant macrophage modules. Gene signatures for macrophages from the study by Xue et al (Reference [28]) were used. This table shows the gene signatures of the most relevant macrophage modules for this study (module number 7, 9, 10, 11, 22, 28, 36, 39, 42, 49). (XLSX 29 kb)

## Abbreviations

COPD: Chronic obstructive pulmonary disease
CT: Computed tomography
GSVA: Gene set variation analysis
NSCLC: Non-small cell lung carcinoma
SEM: Standard error of mean
